# Dung Beetle Optimizer Algorithm and Machine Learning-Based Genome Analysis of *Lactococcus lactis*: Predicting Electronic Sensory Properties of Fermented Milk

**DOI:** 10.3390/foods13131958

**Published:** 2024-06-21

**Authors:** Jinhui Dai, Weicheng Li, Gaifang Dong

**Affiliations:** 1 College of Computer and Information Engineering, Inner Mongolia Agricultural University, Hohhot 010011, China; daijinhui@emails.imau.edu.cn; 2 Inner Mongolia Autonomous Region Key Laboratory of Big Data Research and Application of Agriculture and Animal Husbandry, Hohhot 010011, China; 3 Key Laboratory of Dairy Biotechnology and Engineering (IMAU), Ministry of Education, Inner Mongolia Agricultural University, Hohhot 010018, China; liweicheng0011@163.com; 4 Key Laboratory of Dairy Products Processing, Ministry of Agriculture and Rural Affairs, Inner Mongolia Agricultural University, Hohhot 010018, China; 5 Inner Mongolia Key Laboratory of Dairy Biotechnology and Engineering, Inner Mongolia Agricultural University, Hohhot 010018, China; 6 Collaborative Innovative Center for Lactic Acid Bacteria and Fermented Dairy Products, Ministry of Education, Inner Mongolia Agricultural University, Hohhot 010018, China

**Keywords:** *Lactococcus lactis*, fermented milk, electronic sensory characteristics, DBO optimization algorithm, feature selection, ridge regression

## Abstract

In the global food industry, fermented dairy products are valued for their unique flavors and nutrients. *Lactococcus lactis* is crucial in developing these flavors during fermentation. Meeting diverse consumer flavor preferences requires the careful selection of fermentation agents. Traditional assessment methods are slow, costly, and subjective. Although electronic-nose and -tongue technologies provide objective assessments, they are mostly limited to laboratory environments. Therefore, this study developed a model to predict the electronic sensory characteristics of fermented milk. This model is based on the genomic data of *Lactococcus lactis*, using the DBO (Dung Beetle Optimizer) optimization algorithm combined with 10 different machine learning methods. The research results show that the combination of the DBO optimization algorithm and multi-round feature selection with a ridge regression model significantly improved the performance of the model. In the 10-fold cross-validation, the R^2^ values of all the electronic sensory phenotypes exceeded 0.895, indicating an excellent performance. In addition, a deep analysis of the electronic sensory data revealed an important phenomenon: the correlation between the electronic sensory phenotypes is positively related to the number of features jointly selected. Generally, a higher correlation among the electronic sensory phenotypes corresponds to a greater number of features being jointly selected. Specifically, phenotypes with high correlations exhibit from 2 to 60 times more jointly selected features than those with low correlations. This suggests that our feature selection strategy effectively identifies the key features impacting multiple phenotypes, likely originating from their regulation by similar biological pathways or metabolic processes. Overall, this study proposes a more efficient and cost-effective method for predicting the electronic sensory characteristics of milk fermented by *Lactococcus lactis*. It helps to screen and optimize fermenting agents with desirable flavor characteristics, thereby driving innovation and development in the dairy industry and enhancing the product quality and market competitiveness.

## 1. Introduction

The dairy industry holds a pivotal position in the global food sector, and fermented dairy products like yogurt and cheese are especially favored for their unique flavors and nutritional values [1,2,3]. *Lactococcus lactis*, a crucial fermenting agent in dairy production, plays a key role in the formation of the final product flavor [4]. Flavor, as a key element that attracts consumers to fermented dairy products, is undeniably important [5]. Therefore, to meet the increasingly diverse flavor demands of consumers and to drive the continuous development of the dairy industry, the precise selection and optimization of *Lactococcus lactis* fermentation agents with ideal flavor characteristics are particularly important. This is not only a major technical challenge faced by the dairy industry but is also key to enhancing the product quality and winning market competitiveness.

Traditional methods of flavor assessment are limited due to their long experimental cycles, high costs, and strong dependence on subjective human sensory evaluation systems. The nine-point hedonic scale is one of them [6]. These methods are susceptible to individual differences and perceptual biases, making it difficult to ensure consistent and accurate assessments of the flavor contributions of *Lactococcus lactis* in fermented milk. DeBruyne and Hekmat (2024) explored the effects of adding various functional powders on the survival of probiotics and their flavor attributes in yogurt in their study [7]. Their research used traditional sensory evaluation methods, with 86 participants employing a nine-point hedonic scale to assess the appearance, flavor, texture, and overall acceptance of yogurt. The study found that the treatments with added rice powder scored higher in appearance, flavor, texture, and overall acceptance, indicating that the addition of functional powders could effectively enhance the nutritional value and health benefits of yogurt, while also attracting new consumers. However, despite receiving positive feedback through sensory evaluations, this method is still limited by its subjectivity. The individual differences and cultural backgrounds of the participants might affect their sensory evaluations, potentially leading to inconsistencies in their flavor perception and inaccuracies in the evaluations. Furthermore, sensory evaluation methods typically require a long time and high costs, including the recruitment and training of sensory evaluation panels, as well as multiple rounds of testing to ensure the reliability of results [8].

However, the rapid development of electronic-nose and electronic-tongue technologies has brought revolutionary changes to the flavor assessment of fermented dairy products. These technologies, by simulating the human olfactory and gustatory systems and using arrays of high-sensitivity sensors, can precisely identify and quantify the volatile organic compounds and various taste components in fermented milk samples, providing an objective, real-time, and efficient means for flavor assessment [9]. The electronic nose, with its metal oxide sensors, sharply captures and differentiates the rich aromatic compounds in fermented milk, such as esters, alcohols, and sulfides, which are crucial for the product’s aroma characteristics. The electronic tongue accurately measures the complex taste elements in fermented milk, such as sourness, bitterness, saltiness, umami, and astringency, collectively shaping consumers’ overall sensory experience of the product. Thus, compared to traditional sensory evaluations, electronic-nose and -tongue technologies offer more objective and efficient assessment tools that can quickly differentiate the subtle flavor differences of various fermenting strains. This not only helps optimize fermentation processes, accelerate the development of new products, and reduce production costs, but it also supports the selection of ideal *Lactococcus lactis* fermentation agents and personalized flavor customization, thereby enhancing the overall product quality and market competitiveness.

Fujioka’s (2021) study used electronic-nose technology to assess the aroma intensity of cheese and compared it with sensory evaluation scores [10]. The study found a significant correlation between the measurements of the electronic nose and the sensory evaluation scores for the aroma intensity. Particularly during chewing, a linear relationship was shown between the aroma intensity scores and the measurements from the electronic nose, highlighting the potential of the electronic nose in predicting cheese flavors. In Chi’s (2022) research, the team combined the use of the electronic nose, electronic tongue, and Gas Chromatography–Ion Mobility Spectrometry (GC-IMS) to analyze the impact of key additives during processing on the flavor of infant formula milk [11]. The study demonstrated that the integrated use of the electronic nose and tongue provides an efficient method for flavor assessment, which is significant for the quality control of dairy products. Zeng’s (2023) study quickly predicted the aroma types of different plain yogurts using an electronic nose [12]. The study showed that the sensor values from the electronic nose were highly correlated with the sensory evaluation scores for the aroma types. This result further validates the application value of the electronic nose in rapidly and accurately assessing the aroma types of fermented dairy products. Lee-Rangel’s (2022) study applied electronic-nose technology and HS-SPME/GC-MS to analyze the volatile organic compounds (VOCs) in fresh Mexican cheese made from the milk of two different dairy cattle breeds [13]. The e-nose Cyranose 320 effectively differentiated between cheeses made from Holstein and Jersey cow milk, identifying the key VOCs, such as carboxylic acids, that are essential in the dairy industry. The research highlights the e-nose’s potential for cheese authentication and quality evaluation via the detection of specific aroma compounds linked to different milk sources. Zhang’s (2023) study examined the effects of jujube powder (JP) supplementation on cow’s milk, finding that it enhanced the antioxidant capacity and lactoferrin and IgG levels [14]. Using electronic-nose and GC-MS technology, they observed increased ketones and decreased acids in the JP milk, which altered its flavor profile. The results indicate that JP supplementation can improve milk’s bioactive components and flavor, suggesting its potential for healthier dairy products. Hayashida’s (2023) study employed electronic-tongue technology to evaluate the taste characteristics of cheeses matured with different Koji mold strains and compared them with traditional Camembert cheese [15]. The study revealed significant differences in the acidity, bitterness, astringency, saltiness, and umami richness between the Koji cheeses and commercial cheeses, indicating that by selecting different Koji mold strains, the taste characteristics of cheese can be customized. This study emphasizes the effectiveness of the electronic-tongue system in distinguishing cheese flavors, providing a new technical approach for their precise analysis.

Electronic-nose and -tongue technologies offer relatively objective, novel approaches for evaluating the flavors of foods. Additionally, some electronic noses can perform online sample analysis and rank the results based on criteria such as the level of the quality degradation of food. However, in the flavor assessment of fermented dairy products, their use is mainly confined to laboratory settings and requires intricate sample preparation, equipment calibration, and data analysis. Therefore, they cannot completely replace comprehensive flavor assessments in the early stages of new product development.

**Figure 1:**
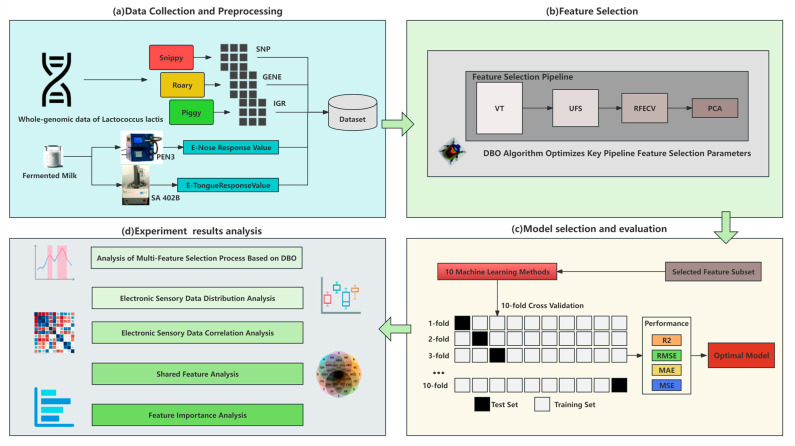
Flowchart of the predictive study of electronic sensory characteristics of *Lactococcus lactis*-fermented milk. This chart illustrates the entire research process from the data collection to the final analysis of the experimental results, including four main stages: (**a**) data collection and preprocessing, (**b**) feature selection, (**c**) model selection and evaluation, and (**d**) analysis of the experimental results.

For this reason, this study utilized machine learning technology, combined with the genomic data of *Lactococcus lactis* and the phenotypic data obtained from the electronic nose and tongue, to build a predictive model to assess the fermentation flavor characteristics of *Lactococcus lactis*. By utilizing feature selection technology based on the Dung Beetle Optimizer algorithm, the efficiency and accuracy of identifying flavor-related features from high-dimensional genomic data were improved, enhancing the model performance and stability. Ten different machine learning methods were applied to verify the impacts of these features, ensuring scientific validity and reliability. An in-depth analysis of electronic sensory data identified shared features across phenotypes and their importance rankings, exploring the potential connections between the genomic information of *Lactococcus lactis* and the flavor of fermented milk. These research findings provide a robust scientific basis for the precise selection and optimization of *Lactococcus lactis* fermentation agents with the ideal flavor characteristics, and they offer significant technical support for the refined development and quality control of fermented milk products. Figure 1 provides a visual representation of this study.

## 2. Materials and Methods

### 2.1. Data Collection of Lactococcus lactis Strains

The 194 strains of *Lactococcus lactis* used in this study, along with their whole-genome data, were sourced from the Key Laboratory of Dairy Biotechnology and Engineering of the Ministry of Education at Inner Mongolia Agricultural University. The response value data for assessing the taste and odor characteristics of fermented milk using electronic-tongue and electronic-nose technologies were also provided by this laboratory. The electronic-tongue technology utilized an SA 402B device manufactured by Insent, Japan, for the data collection. Equipped with 5 taste sensors and 2 reference electrodes, this device accurately measures the various taste sensations in fermented milk, including umami, saltiness, sourness, bitterness, and astringency, as well as the richness, aftertaste bitterness (Aftertaste-B), and aftertaste astringency (Aftertaste-A). The electronic-nose system utilizes a PEN3 device manufactured by Airsense, Germany, to obtain response value data for detecting volatile organic compounds and other odor-active substances in fermented milk. This device incorporates 10 metal oxide sensors; the details of the sensitive substances are outlined in Table 1.

### 2.2. Data Preprocessing

Data preprocessing was a crucial step in this study, aimed at optimizing and standardizing the genomic data of the *Lactococcus lactis* strains and the phenotype data obtained through the electronic tongue and electronic nose, ensuring the accuracy and reliability of the subsequent statistical analysis and machine learning model construction.

Quality control of electronic sensory data: We rigorously reviewed the results of repeated measurements of the same strain in multiple experiments to ensure the accuracy and consistency of the taste and aroma indicators in fermented milk.

Integration of genomic data: In the processing of the genomic data of *Lactococcus lactis*, we first used Snippy [16] to perform whole-genome variation detection on 194 strains of *Lactococcus lactis*, focusing on the analysis of single-nucleotide polymorphisms (SNPs) and insertions/deletions (indels). Then, we analyzed the whole-genome structure of these strains using Roary [17] to construct a matrix of the gene presence and absence, revealing the distribution of core and accessory genes in the genome. Additionally, we used the Piggy [18] to conduct an in-depth analysis of the non-coding regions in the genome, detecting critical variations in the intergenic regions (IGRs) that may affect gene regulation. This series of analyses culminated in the generation of a comprehensive, multidimensional dataset of genetic variations, including an SNP matrix with 107,661 features, a GENE matrix with 49,146 features, and an IGR matrix with 29,301 features. This dataset facilitated an exploration into the relationship between the genomic variations and gene expression changes and how this information collectively influences the fermentation flavor characteristics of *Lactococcus lactis*.

### 2.3. Feature Selection

In this study, the genomic data on *Lactococcus lactis* belong to a high-dimensional dataset with a low sample size, which poses a significant challenge for the standard statistical methods and modern machine learning techniques. To address this challenge, we proposed a multi-round feature selection strategy based on the Dung Beetle Optimizer (DBO) algorithm, which replaces the cumbersome manual parameter tuning and exploration of various feature combinations. This strategy effectively saves experimental time and human resources, and it starts from the direction of finding the global optimal solution. In the process of constructing the prediction model for the fermentation flavor of *Lactococcus lactis*, it conducts the in-depth selection and optimization of the key features, thereby significantly improving the accuracy and reliability of the model.

#### 2.3.1. DBO Optimization Algorithm

The DBO optimization algorithm is a novel swarm intelligence optimization algorithm inspired by the rolling, foraging, stealing, and reproduction behavior patterns of dung beetles, possessing powerful optimization capabilities and fast convergence characteristics [19]. In this study, we applied the DBO algorithm to the feature selection stage to improve the predictive performance of the model.

We configured the DBO algorithm with a population size of 50 beetles, striking a balance between computational efficiency and a thorough exploration of the solution space. A larger population would significantly increase the computational time, while a smaller population might lead to a premature convergence on suboptimal solutions. The roles within the beetle population are distributed as follows: 20% rolling beetles to explore new areas, 40% foraging beetles to refine existing solutions, 20% stealing beetles to integrate successful strategies, and 20% reproducing beetles to maintain diversity. The algorithm is designed to stop after 100 iterations, a criterion set to optimize the balance between the computation time and convergence. This setup efficiently leverages the DBO algorithm’s strengths to optimize the parameters within the feature selection pipeline, ensuring a balanced approach to exploration and exploitation.

#### 2.3.2. Multi-Round Feature Selection Pipeline

Feature selection methods can generally be divided into three types: filter methods, wrapper methods, and embedded methods [20]. Filter methods select features based on statistical tests independently of any learning algorithm; wrapper methods combine the feature selection process with the model-training process, evaluating the quality of the feature subsets based on the predictive performance; embedded methods automatically perform feature selection during the model-training process, such as the regularization operation of Lasso regression. The multi-round feature selection pipeline designed in this study integrates the advantages of the filter, wrapper, and embedded methods. Through a serialized feature selection process, it gradually refines the target feature set, including the following feature selection processes:

VT (removal of low-variance features): By setting a variance threshold, all features with variances lower than this threshold are removed, highlighting the genetic features that show high variability among the strains to emphasize the gene variations and expression patterns potentially significant for the fermentation flavor;

UFS (univariate feature selection): By computing a certain statistical metric for each feature to determine its importance and selecting features based on the feature scores, highly correlated genetic features with the target variable in the fermentation flavor prediction model are retained. In this process, we used the f_regression method as a statistical metric for the univariate feature selection, which is based on the F statistic and is commonly used for regression problems;

RFECV (recursive feature elimination with cross-validation): RFECV is a feature selection method that combines recursive feature elimination with cross-validation. Its main idea is to iteratively construct models and select or exclude features in each iteration based on the model performance while using cross-validation to adjust the number of features. RFECV evaluates the performance of the model with a specific number of features in each iteration and then decides whether to continue the iteration based on a predefined performance metric or feature count. This process helps reduce the number of features and improves the model generalization ability and efficiency by finding the optimal feature subset to achieve the best model performance given a performance metric.

PCA (principal component analysis): Principal component analysis extracts the main features from the data and recombines them into new, fewer dimensions to achieve data dimensionality reduction. The aim is to retain the most critical information while reducing the data complexity for more effective data analysis and model building [21].

#### 2.3.3. Selection of RFECV Estimator

RFECV, as the pivotal component in the multi-round feature selection pipeline, requires the careful selection of the appropriate estimator. This choice significantly impacts the algorithm’s operational efficiency, the accuracy of the feature selection, and the ultimate performance of the model. Moreover, the estimator must be capable of providing an assessment of the feature importance, enabling the determination of the features to be removed in each iteration. To identify the most suitable estimator, we tested various regression algorithms, considering different combinations of feature numbers, and we evaluated the efficiency and effectiveness of each model by calculating the ten-fold cross-validation R^2^ scores of the feature subsets and the time required for the algorithm execution.

#### 2.3.4. Multi-Round Feature Selection Strategy Based on DBO Optimization Algorithm

In this study, we employed the DBO optimization algorithm to adjust the key parameters in the multi-round feature selection process. Our primary goal was to enhance the contribution of the features selected by the feature pipeline to the predictive performance of the model, thereby identifying a concise feature subset that enhances the model accuracy. Table 2 summarizes the parameters involved in the multi-round feature selection pipeline when using the DBO optimization algorithm, with the parameter settings directly impacting the effectiveness and ultimate performance of the model’s feature selection.

**Figure 2:**
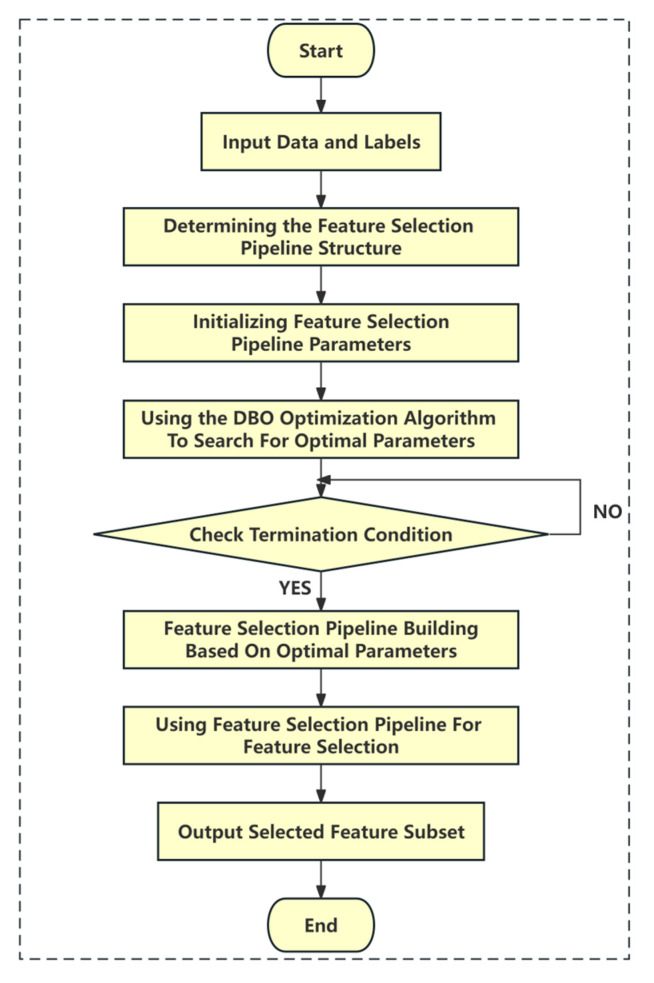
Flowchart of the multi-round feature selection process based on the DBO optimization algorithm. First, initialize the parameters of the feature pipeline and perform DBO optimization to find the optimal parameters. Then, utilize the optimized feature selection pipeline to filter the unimportant features and select the subset of features that contributes the most to the target prediction. The entire process integrates DBO optimization with feature selection techniques to improve accuracy and efficiency in the selection.

By optimizing these parameters through the DBO algorithm, we were able to automate and precisely adjust the feature selection process, effectively screening out key genetic features from high-dimensional genomic datasets. This enhanced the accuracy and stability of the *Lactococcus lactis* fermentation flavor prediction model and provided robust support for the subsequent data analysis and model construction. The flowchart of the multi-round feature selection process based on the DBO optimization algorithm is illustrated in Figure 2.

### 2.4. Establishment and Evaluation of Machine Learning Models

Given the limitation in the number of *Lactococcus lactis* strain samples, this study utilized machine learning techniques to predict the response values of the electronic tongue and electronic nose in *Lactococcus lactis*-fermented milk. Through multiple rounds of feature selection, we isolated a core set of features associated with each flavor phenotype. These features are crucial to the underlying mechanisms that influence the phenotype, significantly improving the predictive accuracy and generalization capability of our model.

Following the application of the DBO optimization algorithm-based feature selection, it became crucial to determine the most effective machine learning algorithms capable of processing the refined feature subsets. Consequently, we evaluated ten classic machine learning regression algorithms, aiming to maximize the R^2^ in predicting the phenotype responses of the electronic-nose and -tongue technologies. The algorithms tested included Ridge regression [22], neural network regression [23], support vector regression [24], random forest regression [25], Lasso regression [26,27], gradient-boosting regression [28], decision tree regression [29], XGBoost regression [30], K-nearest neighbor regression [31], and elastic-net regression [32].

The model performance was evaluated using multiple evaluation criteria, including the coefficient of determination (R^2^), mean absolute error (MAE), mean-squared error (MSE), and root-mean-squared error (RMSE). A higher R^2^ value indicates a better model fit and prediction performance. The MAE and MSE measure the deviation from the predicted true value from the angle of absolute error and square error, respectively. The RMSE is the square root of the MSE. The smaller the value of these indexes, the better the prediction performance. Considering the limitation in the sample size, we adopted a 10-fold cross-validation strategy, where the dataset was randomly divided into 10 parts, each serving as a test set in turn, with the remaining parts combined as the training set, for 10 rounds of training and testing. This ensured that each sample participated in the testing process, effectively preventing model evaluation bias and improving the robustness and representativeness of the evaluation results. During the cross-validation, the mean of each evaluation metric (R^2^, MAE, MSE, RMSE) across all iterations was calculated to obtain the stable and representative performance evaluation values of the model on the entire dataset.

By comprehensively using these evaluation metrics, we were not only able to accurately quantify and compare the performances of different models, but we also gained insight into the multidimensional performances of the models in terms of accuracy, stability, and interpretability. This approach contributes to providing robust support for optimizing the selection process of *Lactococcus lactis* strains and precisely controlling the flavor quality of fermented milk products.

## 3. Results and Discussion

### 3.1. Result of Feature Selection

#### 3.1.1. Selection and Evaluation of RFECV Estimator

In the feature selection pipeline, RFECV plays a crucial role. Choosing the appropriate estimator is essential to ensure the effectiveness of the feature selection. This study conducted a series of experiments to determine the most suitable estimator. The experiments were conducted around different feature numbers (from 1000 to 5000) to evaluate the performances and efficiencies of various regression algorithms. When evaluating the performances of the estimators, we used the R^2^ value. A higher R^2^ value closer to 1 indicates a better fit of the model to the data, while a negative R^2^ value indicates a poor model fit. Efficiency in the feature selection process is crucial when dealing with high-dimensional feature datasets, so we also assessed the computation times of the estimators. The experimental results are shown in Table 3 and Table 4.

The results show differences in the efficiencies and performances among the different estimators. We ultimately chose ridge regression as the estimator for the RFECV, primarily based on the following considerations.

Firstly, ridge regression demonstrates an efficient computational capability in high-dimensional data environments. Compared to other complex models, such as gradient-boosting regression and random forest regression, ridge regression exhibits a higher computational efficiency and execution speed. Secondly, ridge regression maintains high R^2^ scores under different feature numbers, indicating its effective use of the selected feature set for accurate prediction under RFECV assistance. Additionally, ridge regression effectively addresses multicollinearity issues among features through L2 regularization, enhancing the robustness of datasets with high correlation. Moreover, as a linear model, ridge regression coefficients directly reflect the influences of the features on the target variable, facilitating the interpretation of the impact of each feature on the fermented milk flavor and providing strong interpretability. Finally, the ridge regression parameters are relatively simple, mainly consisting of adjusting the regularization strength, simplifying the model-training and parameter-tuning processes, and improving the efficiency of the feature selection. According to Buteneers, the optimization of the regularization parameters and feature selection for ridge regression significantly improves the computational efficiency and model robustness when dealing with large datasets [33].

Furthermore, the experiments also found that as the feature dimension increases, the computational cost of the RFECV significantly increases, but the improvement in the model prediction performance is limited, sometimes even decreasing. This is evident from the data presented in Table 3 and Table 4. Table 3 shows the computation times of the different estimators for feature numbers ranging from 1000 to 5000. For instance, the computation time for the ridge estimator increases from 8.3 s at 1000 features to 82.2 s at 5000 features. Similarly, for the RFR estimator, the computation time jumps from 855.4 s to 17,319.1 s as the feature number increases from 1000 to 5000. This clearly illustrates the significant rise in the computational cost with increasing feature dimensions. Table 4 compares the cross-validation performances (R^2^ values) of the different estimators over the same range of feature numbers. It can be observed that while the ridge estimator’s R^2^ value improves initially (from 0.320 at 1000 features to 0.821 at 4000 features), it decreases to 0.573 at 5000 features. This pattern is also observed with the other estimators, such as SVMR, for which the R^2^ value increases from 0.424 at 1000 features to 0.709 at 3000 features, but it then drops to 0.441 at 5000 features. Therefore, preliminary feature screening before applying RFECV is crucial to alleviate the computational burden and improve the prediction accuracy and stability, allowing the RFECV to focus more on identifying and evaluating the features that substantially impact the prediction targets, thereby optimizing the efficiency and effectiveness of the overall feature selection process.

#### 3.1.2. Effectiveness of Multi-Round Feature Selection Method

**Figure 3:**
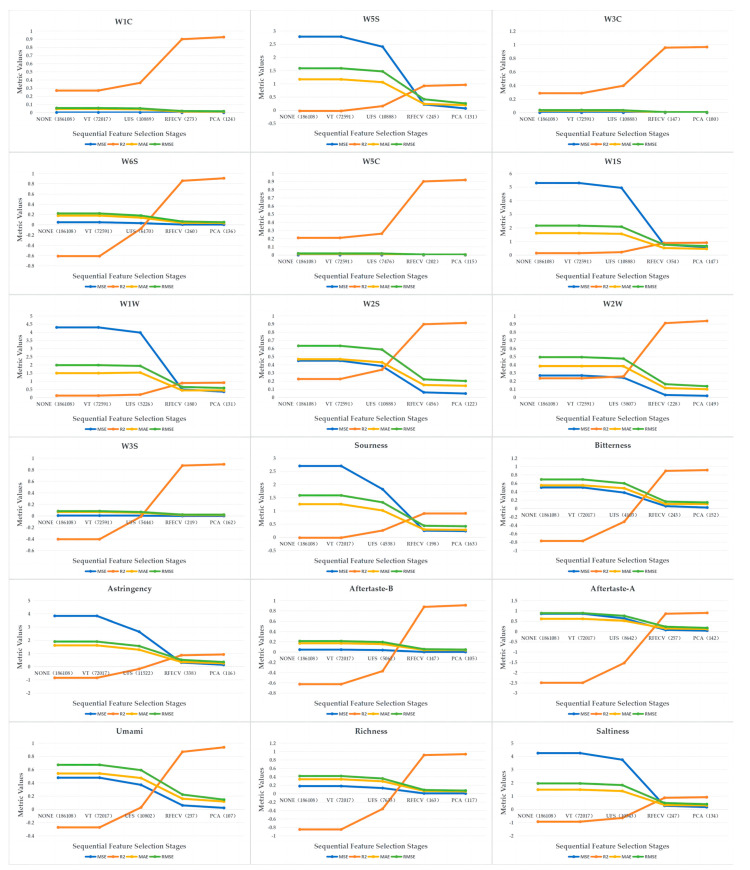
Effectiveness of multi-stage feature selection. NONE (did not use any feature selection); VT (removal of low-variance features); UFS (univariate variable feature selection); RFECV (recursive feature elimination and cross-validation); PCA (principal component analysis); numbers in parentheses represent the current feature dimension. Note: the vertical-axis ranges are different for each graph to accurately reflect the performance improvements at each feature selection stage.

Figure 3 illustrates how the predictive performance of each phenotype changed after the different feature selection stages. Taking the W1C phenotype of the electronic tongue as an example, a large number of redundant features were successfully eliminated in the removal of low-variance feature stage, significantly reducing the data dimensionality. Subsequently, in the univariate feature selection stage, we accurately identified features with lower correlations with the target variable, further reducing the data dimensionality and improving the R^2^ score. Next, in the RFECV stage, we conducted an in-depth selection of the remaining features, further optimizing the feature set and significantly improving the model performance. Finally, in the PCA stage, we further refined the key information, which resulted in a new high for the R^2^ score.

Overall, our multi-round feature selection process, spanning from removing low-variance features to PCA, offers a logical and highly effective methodological approach. The experimental results vividly showcase its prowess in removing redundancy, selecting pivotal features, and leveraging the data structure for an enhanced predictive performance. This underlines the scientific and practical efficacy of our approach.

### 3.2. Performances of Different Algorithms with Selected Feature Subsets

**Figure 4:**
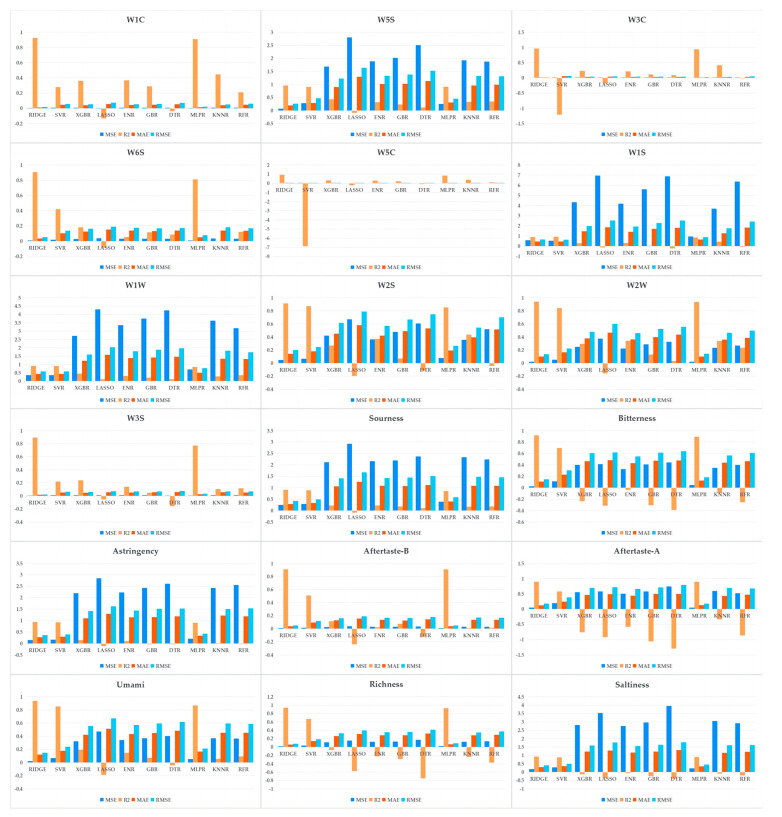
Performance comparison diagram of different machine learning algorithms. Note: the vertical-axis ranges vary across graphs to accurately reflect the performance differences among the different machine learning algorithms.

After employing the DBO optimization algorithm-based feature selection, it became imperative to identify the most effective machine learning algorithm capable of handling the refined feature subsets. Accordingly, we evaluated ten different machine learning algorithms for their abilities to predict the phenotype responses of the electronic-nose and electronic-tongue technologies. The primary goal of this analysis was to assess and compare the accuracies of these algorithms in capturing the electronic sensory characteristics of fermented milk. This comparative approach was pivotal in selecting the optimal algorithm that best addresses the challenges of electronic sensory prediction tasks. Given the scale of the dataset, we adopted a rigorous 10-fold cross-validation strategy to ensure the reliability and effectiveness of the model evaluation. We demonstrated the comprehensive performance of each algorithm in terms of its prediction accuracy, stability, and model-fitting quality using multiple key statistical metrics, including the mean-squared error (MSE), coefficient of determination (R^2^), root-mean-squared error (RMSE), and mean absolute error (MAE). The results, as shown in Figure 4 and Table 5, revealed that the ridge regression performed excellently in most phenotype predictions, with R^2^ values generally exceeding 0.8951. Although the support vector machine regression exhibited the best performance in the W1S phenotype, with an R^2^ of 0.9209, the ridge regression closely followed with an R^2^ of 0.9173. This result underscores the effectiveness and reliability of integrating the DBO-based multi-round feature selection method with ridge regression. This integration significantly enhances our ability to accurately predict the electronic sensory characteristics of fermented dairy products. Such robust predictive capabilities establish a solid scientific foundation for the precise selection and optimization of *Lactococcus lactis* fermentation agents with the ideal flavor characteristics. Moreover, they offer substantial technical support for the refined development and quality control of fermented milk products.

### 3.3. Sensory Characteristic Analysis and Correlation Study of Lactococcus lactis-Fermented Milk

#### 3.3.1. Distribution Analysis of Electronic Sensing Data of *Lactococcus lactis*-Fermented Milk

**Figure 5:**
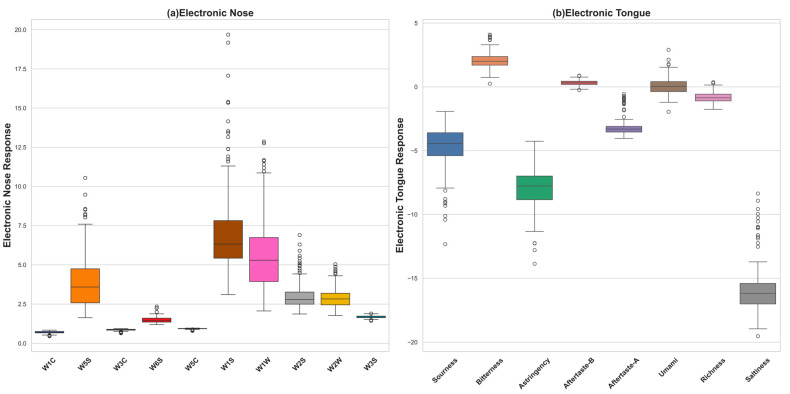
Box diagrams of distributions of electronic sensory characteristics of *Lactococcus lactis* fermented milk.

We conducted a distribution analysis of electronic sensory data for *Lactococcus lactis*-fermented milk and visually displayed it through the box plots in Figure 5. For the electronic-nose data, W5S, W1S, and W1W showed large variation ranges, indicating significant differences in these odor indicators among the samples. Specifically, W1S exhibited the broadest range, from about 3 to just below 20, demonstrating the greatest variability. In comparison, W1W and W5S showed narrower ranges from 2 to 12 and from 2 to over 10, respectively, both less variable than W1S but still displaying significant differences among the sample types. Conversely, the response values for W1C, W3C, W6S, W5C, and W3S were more concentrated, suggesting similarity among the samples in these odor indicators. Regarding the distribution of the response values for the electronic tongue, we observed that sourness, astringency, and saltiness exhibited more scattered distributions, reflecting significant differences in these taste indicators among the samples. However, the distributions of the other taste indicators were relatively centralized, indicating similarity among the samples in these taste indicators. The presence of outliers may suggest the uniqueness of certain strains of *Lactococcus lactis* in the flavor performance.

Overall, the data from the electronic nose and electronic tongue reveal differences in the odor and taste indicators in *Lactococcus lactis*-fermented milk, which may originate from differences in the metabolic characteristics, growth rates, and genetic backgrounds of the different strains, leading to changes in the flavor components [34]. Those phenotypes that are more similar may point to similar fermentation characteristics or shared flavor attributes among *Lactococcus lactis*. The study by Gutiérrez-Méndez (2008) noted that the specific aroma production capabilities of individual *Lactococcus lactis* strains are not directly linked to their sources of isolation [35]. Instead, the aroma generation is primarily driven by the catabolism of amino acids. This indicates that the metabolic processes, especially those involved in amino acid breakdown, play a crucial role in determining the flavor profiles produced by these strains. This suggests that strains sharing similar phenotypic traits, particularly in terms of their metabolic pathways for amino acid catabolism, are likely to produce similar flavor profiles.

#### 3.3.2. Correlation Analysis of Electronic Sensory Data for *Lactococcus lactis*-Fermented Milk

**Figure 6:**
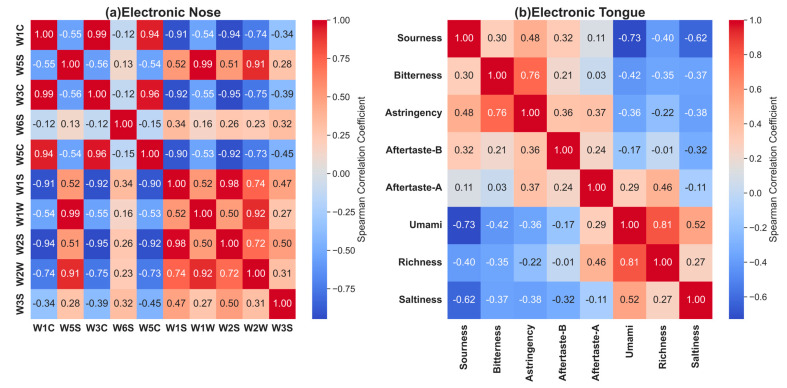
Spearman correlation heatmaps for electronic-nose and -tongue responses.

In this section, we conduct a correlation analysis of electronic sensory data for *Lactococcus lactis*-fermented milk using the Spearman correlation coefficient to reveal the inherent relationships between the taste characteristics and electronic-nose and -tongue detection data. The Spearman correlation is a non-parametric statistic that is suitable for assessing monotonic relationships between variables, regardless of the linearity or normality of the data distribution [36]. The results are visually presented in Figure 6 as Spearman correlation heatmaps.

In the correlation heatmap for the electronic nose, the positive correlation between W1C and W3C and W5C may be related to their sensitivities to aromatic components. Conversely, the negative correlation between W1C and W1S and W2S may indicate changes in the concentration of aromatic components, which have opposite effects on methane and ethanol production. The significant positive correlation between W5S and W1W and W2W suggests that nitrogen compounds have similar response patterns to those of sulfides and organic sulfides, indicating a common source or the interrelated chemical reactions of these odor molecules in *Lactococcus lactis*-fermented milk. This correlation analysis helps understand and regulate the key aroma components of fermented milk.

In the correlation heatmap for the electronic tongue, we observed a clear negative correlation between sourness and umami, richness, and saltiness. This indicates that as the sourness increases, the perceived intensities of these taste characteristics decrease, suggesting that the sourness level may be a key factor affecting the overall taste characteristics of fermented milk. The positive correlation between bitterness and astringency suggests that they may share taste sources or enhance each other in terms of perception. Furthermore, the positive correlation between umami and richness emphasizes the importance of umami for the taste experience and the formation of persistent flavor.

Through the sensory characteristic analysis and correlation study of *Lactococcus lactis*-fermented milk, we can gain a deep understanding of the unique differences and commonalities in the aromas and tastes exhibited by fermented milk prepared from different strains of *Lactococcus lactis*. This not only promotes the evaluation of the *Lactococcus lactis*-fermented flavor characteristics but also enhances the understanding of the mechanisms of various *Lactococcus lactis* strains in flavor formation. These analyses demonstrate the potential application value of electronic sensory technology in the development and quality control of fermented milk products, showcasing its enormous potential as a tool for food industry quality monitoring and flavor optimization.

### 3.4. Analysis of Shared Features of Electronic Sensory Sensors

**Figure 7:**
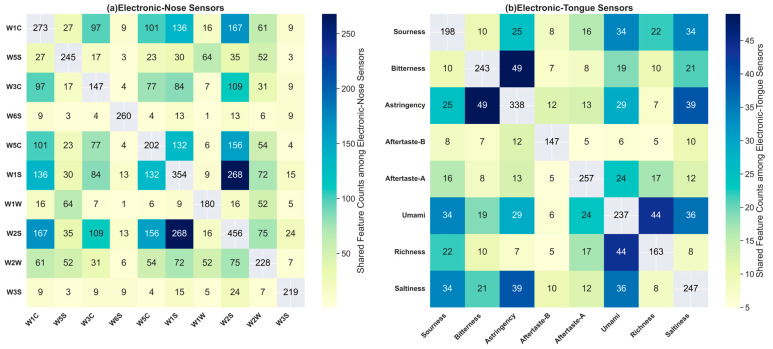
Heatmaps of the numbers of shared original features of the electronic-nose and electronic-tongue sensors.

In our in-depth analysis of *Lactococcus lactis* genomic data, we identified some key trends based on the number of shared original features of the electronic sensory sensors presented in Figure 7 and the correlation heatmap of electronic sensory data shown in Figure 6. This study revealed a clear positive correlation between the correlation among the electronic sensory phenotypes and the number of features selected through feature engineering. Specifically, phenotypes with high correlations exhibit from 2 to 60 times more jointly selected features than those with low correlations. For instance, the correlation between umami and richness is highly significant at 0.81, with the highest number of shared features at 44. This is followed by the correlation between umami and sourness, which is −0.73, with 34 shared features. In contrast, the correlation between umami and Aftertaste-B is only −0.17, with a minimal number of shared features at 6.

However, for phenotypes with lower correlations, the situation is different. The number of shared features between them is significantly reduced, suggesting that these phenotypes may be controlled by different biological mechanisms or influencing factors, resulting in a more unique distribution and expression of the features selected for each phenotype.

When analyzing the phenotypes obtained from the electronic nose, the above trends are more pronounced. Taking the W1S phenotype as an example, it exhibits high correlations with the W1C, W3C, W5C, and W2S phenotypes, with corresponding numbers of shared features of 136, 84, 132, and 268, respectively. In contrast, the W6S phenotype shows lower correlations with all the other phenotypes, with a correspondingly limited number of shared features. This phenomenon suggests that the ability of *Lactococcus lactis* to produce specific volatile molecules in fermented milk is likely tightly regulated by specific genetic information, and the gene expression patterns corresponding to different phenotypes determine their differences in volatile-compound synthesis.

Furthermore, it was observed that the number of shared features between the phenotypes detected by the electronic nose generally exceeded those detected by the electronic tongue. This may indicate that during the fermentation process of *Lactococcus lactis*, there may be a core set of genetic information that plays a dominant role in the generation of gases or volatile organic compounds in the fermented milk, and this regulatory effect is more concentrated compared to the formation of the flavor components in the fermented milk. These observed patterns provide important clues for the application of electronic sensory technology in the development and quality control of fermented milk products, although further research is required to validate these findings and explore their biological significance.

Our research demonstrates the effectiveness of our feature selection method at identifying the key features that have significant impacts on multiple phenotypes. The increase in shared features implies that these phenotypes may be influenced by similar biological pathways or metabolic processes, underscoring the potential of our approach in unraveling the complex metabolic pathways affecting flavor characteristics.

### 3.5. Feature Importance Analysis

In this study, we combined PCA with ridge regression models to evaluate the impacts of the original features on the prediction targets. Firstly, PCA was applied to reduce the dimensionality of the refined original-feature set, extracting the key information and quantifying the contribution of each feature to the principal components (loadings) [37]. Next, the principal components were used as the inputs to construct the ridge regression models, where the coefficients revealed the direct effects of each principal component on the prediction results. To further understand the indirect effects of the original features, we combined PCA loadings with ridge regression coefficients to calculate the total contribution of each feature to the prediction target through all the principal components, considering their positive and negative influences. This method effectively identified the features with the greatest impacts on the prediction results, enhancing the feature selection and model interpretability.

**Figure 8:**
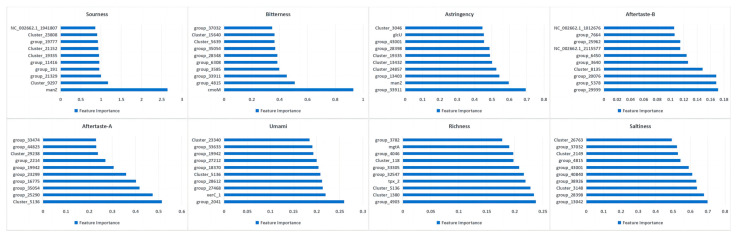
Top 10 rankings of feature importance for electronic-tongue phenotypes.

For the electronic sensory phenotypes of *Lactococcus lactis*, we applied a combination of PCA and ridge regression to analyze their feature importance. For brevity, this paper presents only the top 10 feature importance rankings for all the phenotypes of the electronic tongue, as shown in Figure 8.

## 4. Conclusions

In this study, we successfully utilized genomic data from *Lactococcus lactis* to establish a predictive model for the electronic sensory characteristics of fermented milk. This model integrates multi-round feature selection optimized by the Dung Beetle Optimization algorithm with ridge regression, significantly enhancing the performance. In the 10-fold cross-validation, the R^2^ values for all the electronic sensory phenotypes exceeded 0.895, demonstrating the model’s exceptional performance.

Through an analysis of electronic-nose and -tongue data, we identified a strong positive correlation between the number of jointly selected features and the correlation among the electronic sensory phenotypes, with the highly correlated phenotypes having from 2 to 60 times more shared features than the less correlated ones. This indicates that our feature selection strategy effectively pinpoints the critical features impacting multiple phenotypes, likely governed by similar biological pathways or metabolic processes. The assessment of the feature importance also sheds light on each feature’s impact on the model performance, which is essential for elucidating the biological mechanisms of *Lactococcus lactis* in flavor formation. This approach enables the precise identification and utilization of genetic information crucial for optimizing the screening of and improvement in *Lactococcus lactis* fermenting agents.

In summary, this study presents a more efficient and cost-effective method for predicting the electronic sensory characteristics of *Lactococcus lactis*-fermented milk, aiding in the selection and optimization of fermenting agents with the ideal flavor profiles. This not only advances innovation and development in the dairy industry but also enhances the product quality and market competitiveness. Additionally, we have successfully extended this method to other phenotypes of *Lactococcus lactis*-fermented milk, such as the viscosity and water-holding capacity, although the application to unknown species and other characteristics necessitates further exploration in future studies. Future research could further explore various types of machine learning models and optimization algorithms to enhance the model’s generalizability and practicality in real-world applications.

## Figures and Tables

**Table 1:** Description of electronic-nose sensing performance.

Sensor	Main Performance
W1C	Aromatic constituent
W5S	Nitride oxides
W3C	Ammonia and aromatic constituent
W6S	Hydrogen
W5C	Alkanes and aromatic constituent
W1S	Methane
W1W	Sulfide
W2S	Alcohol
W2W	Aroma constituent and organic sulfur compounds
W3S	Alkanes

**Table 2:** Key parameters and descriptions of the multi-round feature selection pipeline.

Feature Selection Stage	Parameter	Description	Parameter Value Range
VT (removal of low-variance features)	Variance threshold (threshold)	Set a threshold to remove all features with variances below this value, selecting features that significantly impact the model’s predictive ability.	The threshold range is from 0 to 0.0001 (carefully chosen to avoid filtering out too many key features).
UFS (univariate feature selection)	Percentage of features retained (percentile)	Determine the importance of each feature using the f_regression score and retain the most important features based on the set percentage.	Percentile ranges from 1 to 100 (adjustable based on results).
RFECV (recursive feature elimination with cross-validation)	Step (step)	Define the number of features removed in each iteration.	Step ranges from 1 to 10 (adjustable based on results).
	Minimum number of features to select (min_features_to_select)	Set the minimum number of features to keep in the model.	Min_features_to_select ranges from 100 to 300 (adjustable based on results).
	Scoring criterion (scoring)	Method used to evaluate the performance of the feature set.	Scoring options include R2, MSE, RMSE, MAE.
	Estimator parameters (estimator)	Specific configurations for the base model used in the RFECV process.	The range of parameters for the estimator depends on its specific configurations.
PCA (principal component analysis)	Number of principal components retained (n_components)	Set the target number of dimensions after PCA reduction to simplify the dataset while retaining the most important information.	The value of n_components must be less than the total number of samples and the current number of remaining features.

**Table 3:** Comparison of computation times of different estimators with 1000–5000 feature numbers.

Number of Features	Ridge	GBR	DTR	RFR	SVMR	LASSO	ElasticNet
1000	8.3 s	289.3 s	7.5 s	855.4 s	30.5 s	6.0 s	**5.9 s**
2000	18.5 s	869.1 s	41.2 s	2846.2 s	104.5 s	16.2 s	**16.0 s**
3000	35.6 s	1952.1 s	84.4 s	5955.5 s	235.9 s	30.3 s	**30.6 s**
4000	**56.9 s**	4552.7 s	210.0 s	8675.2 s	363.6 s	61.0 s	60.2 s
5000	**82.2 s**	4160.4 s	270.7 s	17,319.1 s	1549.9 s	168.7 s	181.8 s

Note: Bold values indicate the minimum computation times for each row.

**Table 4:** Comparison of cross-validation performances of different estimators with 1000–5000 feature numbers.

Number of Features	Ridge	GBR	DTR	RFR	SVMR	LASSO	ElasticNet
1000	0.320	0.217	−0.263	0.113	**0.424**	−0.085	−0.085
2000	0.554	0.189	−0.357	0.129	**0.627**	−0.085	−0.085
3000	0.644	0.185	−0.224	0.107	**0.709**	−0.085	−0.085
4000	**0.821**	0.356	−0.479	0.128	0.445	−0.085	−0.085
5000	**0.573**	0.205	−0.248	0.130	0.441	−0.085	−0.085

Note: Bold values indicate the highest cross-validation performances for each row.

**Table 5:** Optimal models and scores for electronic-tongue and electronic-nose phenotypes.

Sensor	Optimal Model	MSE	R^2^	MAE	RMSE
Sourness	Ridge Regression	0.242228	0.912875	0.28871	0.423037
Bitterness	Ridge Regression	0.025833	0.917565	0.110485	0.147901
Astringency	Ridge Regression	0.147368	0.932897	0.271402	0.359789
Aftertaste-B	Ridge Regression	0.002615	0.910447	0.038269	0.049111
Aftertaste-A	Ridge Regression	0.047507	0.901767	0.125836	0.180393
Umami	Ridge Regression	0.023892	0.938772	0.119695	0.147940
Richness	Ridge Regression	0.006252	0.940943	0.059736	0.076797
Saltiness	Ridge Regression	0.178090	0.926914	0.308536	0.398723
W1C	Ridge Regression	0.000358	0.926907	0.012767	0.018174
W5S	Ridge Regression	0.074252	0.966470	0.199094	0.261329
W3C	Ridge Regression	0.000070	0.966785	0.006561	0.008178
W6S	Ridge Regression	0.003801	0.908075	0.035363	0.05207
W5C	Ridge Regression	0.000057	0.921548	0.004507	0.006732
W1S	Support Vector Regression	0.544779	0.920901	0.461143	0.647390
W1W	Ridge Regression	0.362764	0.917251	0.439737	0.591199
W2S	Ridge Regression	0.049163	0.915713	0.144283	0.203081
W2W	Ridge Regression	0.020899	0.939947	0.101685	0.138290
W3S	Ridge Regression	0.000566	0.895126	0.016934	0.022449

## Data Availability

The data presented in this study are available upon request from the corresponding author due to the policies of the funding agency and authors’ institutions.
